# Supplementary material for: Collaborative intelligence in AI: Evaluating the performance of a council of AIs on the USMLE
Source: PLOS Digit Health. 2025 Oct 9;4(10):e0000787. doi: 10.1371/journal.pdig.0000787 (PMC12510544; doi:10.1371/journal.pdig.0000787)
Supplement: S2 File — (ZIP) [file pdig.0000787.s002.zip › S2 Data Raw data files containing unprocessed question inputs and LLM outputs including transcripts of deliberations/STEP3_Question-93-1_Type-MC-NJ.docx]

**Facilitator** **[Round 1]:** A 24-year-old recent college graduate comes to the office because of a 3-year history of increased frequency of urination and occasional incontinence whenever she travels. She says the urinary symptoms typically begin a few days before she is going to fly and they stop the day after she arrives at her destination. She says she is anxious about flying. She reports having had similar but milder symptoms prior to examinations before graduating. Medical history is otherwise unremarkable. Her only medication is an oral contraceptive. Vital signs are normal. Physical examination discloses no abnormalities. Urinalysis and urine culture are normal. Which of the following is the most appropriate next step? (A) Recommend behavioral therapy (B) Recommend psychoanalytic psychotherapy (C) Recommend that the patient avoid any stressful activities that cause the problem (D) Review the patient's sexual history (E) Reassure the patient that her symptoms will resolve in time

**AI_C:** RESTATING THE QUESTION: The question presents a case of a 24-year-old woman who manifests increased frequency of urination and occasional incontinence in relation to travel, particularly flying, associated with associated anxiety. This is an interesting case as the patient's symptoms seem closely linked to periods of stress or anticipated stress. The question asks what should be the next appropriate step in managing this patient, given that all her medical evaluations have come back normal and she doesn't seem to demonstrate other obvious pathological conditions. PLANNING: Various assumptions that could be considered here include the fact that stress triggers her symptoms, the symptoms are caused by an undetected medical condition, or they are a psycho-somatic manifestation. As the physical examinations and urinalysis have come back normal, it is less likely that this is caused by a physical condition, leaving us with the possibility of a stress-related psycho-somatic manifestation. CLINICAL REASONING: Given that her symptoms seem to correlate with periods of stress or anxiety (traveling, examinations), a psychological etiology seems likely. In the absence of identifiable physical abnormalities, disorders like overactive bladder and urgent urinary incontinence could be considered, but these are usually unresponsive to symptomatic change after the stressor has passed. Therefore, her symptoms might be a manifestation of her body's reaction to her stress or anxiety. CONCLUDING: Cognitive-behavioral therapy (CBT) is a type of psycho-therapeutic treatment that helps patients understand the thoughts and feelings that influence behaviors. CBT is commonly used to treat a wide range of disorders including phobias, addiction, depression, anxiety, etc. It has also been used effectively in treating urinary issues related to stress and anxiety. RESPONSE: Given the correlation with her periods of anxiety and stress and the absence of identifiable physical conditions, the most appropriate action in this case would be (A) Recommend behavioral therapy. This could help the patient develop strategies to manage her anxiety around flying and potentially reduce her symptoms.

**AI_E:** 1) RESTATING THE QUESTION: The query at hand relates to the case of a 24-year-old woman who has a history of increased urination and occasional incontinence, particularly when she travels. These urinary symptoms seem to be linked with the anxiety she experiences before flying and were also observed before her exams. Despite these symptoms, her medical history, physical examination, and urinalysis are all standard. The question aims to identify the most appropriate next course of action. 2) PLANNING: The pattern suggests that her urinary symptoms could be associated with anxiety, mainly when heightened by certain situations like traveling or examinations. Psychiatrists consider this as a physiological response to psychological stress. Whether these symptoms require medical intervention or the extent to which they can be managed by the patient is an assumption that can be evaluated. Alternate assumptions include: - If her anxiety and consequent urinary symptoms severely impact her quality of life, she may benefit from some form of therapy (either behavioural or psychoanalytic psychotherapy). - Alternatively, reducing exposure to stressful situations, like avoiding the travel or exams that seem to trigger these symptoms, might alleviate them. - Other aspects of her sexual history or the impacts of her contraceptive medication might be contributing to her urinary symptoms. Given these assumptions, evaluating them against the symptom severity, frequency, and the individual's overall well-being should provide a clinically-prioritized direction. 3) CLINICAL REASONING: As the woman's symptoms seem to be primarily linked to stressful situations and they remit soon after the stressor is removed, the first assumption seems plausible. Symptoms being triggered by stress, and specifically anxiety, makes it less likely the symptoms are due to influences from her sexual history or contraceptive medication. As the symptoms are recurrent, it's not wise to assume that they'll resolve over time without therapeutic intervention. Lastly, encouraging her to avoid all stressful situations may not be practical and does not offer a constructive solution to manage her apparent anxiety disorder. 4) CONCLUDING: Taking this all into account, the best course of action would be to engage the woman in a therapy that helps manage her stress-induced symptoms. As behavioral therapy often equips individuals with effective coping strategies to manage specific stressors, this approach could be an appropriate intervention. 5) RESPONSE: Considering the specifics of her condition, the most appropriate next step would be to (A) Recommend behavioral therapy.

**AI_B:** (1) RESTATING THE QUESTION: I understand that a 24-year-old woman has been experiencing an increased frequency of urination and occasional incontinence linked with travel and related anxiety. This pattern also held true during her college examination days, though to a lesser intensity. She has no remarkable medical history, takes an oral contraceptive, has normal vital signs, and all physical tests, including a urinalysis and urine culture, have returned normal results. Increased frequency of urination is technically known as 'Polyuria', and 'Incontinence' essentially refers to the loss of bladder control resulting in unwanted leakage of urine. This woman's symptoms being triggered by stressful events like travel and exams point towards a possible psychogenic cause. (2) PLANNING: The primary assumptions here could be underlying physiological issues like urinary tract infection, diabetes, cystitis, kidney problems -- but these seem unlikely given her normal medical tests. The other assumption could be a psychological or stress-related issue resulting in these symptoms. It's reasonable to prioritize this second assumption since the onset of symptoms tends to correlate with her stress levels. (3) CLINICAL REASONING: In situations like this, where physiological factors have been ruled out, it's highly likely that the symptoms are psychogenic. This refers to physical symptoms that can't be traced back to organic disease but are instead related to psychological factors. Considering this young woman's symptoms show up around times of stress (travel, exams), this could point towards a diagnosis of Stress-Induced Urinary Incontinence, a subclass of Functional Urinary Incontinence. Stress-Induced Urinary Incontinence manifests itself via physical symptoms under periods of acute psychological stress. (4) CONCLUDING: Given this woman's otherwise healthy history, normal medical tests, and symptoms correlating with specific moments of acute stress, it's crucial to address the psychological aspects and seek interventions aimed at reducing stress and anxiety, as well as teaching her strategies to manage her urinary symptoms. (5) RESPONSE: Therefore, given the likely psychogenic cause, the most appropriate next step in managing this patient's symptoms would be (A) Recommend behavioral therapy. Behavioral therapy could equip her with techniques to effectively manage her stress levels and related urinary symptoms.

**AI_D:** RESTATING THE QUESTION: The question relates to a 24-year-old woman experiencing an increase in urination frequency and occasional incontinence, notably before she is set to fly and the symptoms ceasing post-arrival. These symptoms seem to be triggered by stress, evident from their onset before examinations as well. Given the context, her medical history, physical examination, laboratory tests are unremarkable except for her oral contraceptive usage. Her body vitals are generally within normal parameters. The question asks what would be the correct next course of action given her situation. PLANNING: Initial assumptions might revolve around a possible physiological issue resulting in the increased frequency, however, given her medical history and lack of abnormal findings, it seems likely the issue is psychological. The link between her symptoms and stressful situations suggests anxiety and potential coping mechanisms are worth investigating. While all the presented next steps could potentially address her issues, focusing on those that directly address her stress and its manifestation would be clinically prioritized. CLINICAL REASONING: The onset of this woman's symptoms appears to coincide with periods of anxiety and stress, such as flying and examinations. The core issue causing these symptoms appears to be the underlying anxiety rather than any physical abnormality. Her urinary symptoms could be considered a psychogenic polydipsia, a condition where increased fluid intake and urination are driven by psychological factors. Considering this, the next steps should revolve around addressing her anxiety and how it manifests. It's also worth noting her use of oral contraceptives which is unlikely to be related given the situational onset of symptoms but still valuable to acknowledge. CONCLUDING: Considering the context, despite all potential treatments available, the most appropriate course of action to suggest would be one that allows her to manage or alleviate her anxiety and its symptoms, especially before situations where she knows she'll be anxious or stressed. Given these, psychotherapy, behavioral therapy, stress avoidance can be all potential options. However, it's crucial the recommended choice is realistic and allows for a long-term strategy for managing her symptoms. RESPONSE: Accordingly, the most appropriate next step would be (A) Recommend behavioral therapy. This intervention can equip her with the necessary strategies and techniques to help manage her symptoms and stress. It's a more direct approach in handling her symptoms compared to avoiding stress or just reassuring her about the resolution of symptoms. Thus, making it a priority, clinically speaking.

**AI_A:** RESTATING THE QUESTION: This case presents a young woman who has experienced increased urinary frequency and instances of urine leakage during periods of travel, specifically when she flies. The symptoms appear to be stress- or anxiety-related as she reports onset prior to flying or examinations. There is no medical history of note, and her only medication is birth control. All physical findings and laboratory results are within normal limits. We are tasked with identifying the best subsequent step in managing this case, offering a range of possible interventions from behavioral therapy, psychoanalytic psychotherapy, activity modification, reviewing her sexual history, or providing patient reassurance. PLANNING: The patient's urinary symptoms appear to be related to stressful events (like flying or taking exams) and don't seem to have an underlying physical cause, given her normal physical and lab findings. This pattern suggests that the urinary symptoms may be a physical manifestation of an anxiety disorder. Several approaches can be taken to manage this condition, such as behavioral therapy, psychoanalytic psychotherapy, reassurance, or avoiding stress triggers. Reviewing the patient's sexual history is unlikely to provide insights into her symptoms, given the context. The emphasis should be on managing the anxiety, which appears to be the primary contributor to her urinary symptoms. CLINICAL REASONING: The essential element to acknowledge in the clinical reasoning of this case is the stress or anxiety preceding the occurrence of symptoms. This temporal relationship strongly implies a psychosomatic component to the urinary issues. Psychosomatic disorders are conditions in which psychological stresses adversely affect physiological (somatic) functioning to the point of distress. Hence by targeting the anxiety, we can likely alleviate the secondary symptoms. Though avoidance of stress is an attractive idea, it may not be practical or feasible especially considering she seems to experience these symptoms in common stress-inducing situations like examinations and travel. Additionally, simply reassuring the patient without offering any substantial help may lead the patient to feel unheard or invalidated. The remaining options are either psychoanalytic psychotherapy or behavioral therapy. Psychoanalytic psychotherapy is a therapeutic process which helps patients understand and resolve their problems by increasing awareness of their inner world and its influence over relationships both past and present. On the other hand, behavioral therapy employs techniques to stop or change maladaptive behaviors. Given the link between her symptoms and anxiety, a treatment approach aimed at teaching the patient skills to manage her fears such as cognitive-behavioral therapy (CBT), is likely to be more beneficial than trying to delve into potentially deeper unconscious conflicts. CONCLUDING: Based on the observation that this individual's symptoms flares up in situations related to anxiety, and considering that her urinary symptoms disrupt her life and seem to be closely tied with her emotional state, the most beneficial approach to managing her symptoms would seem to be offering a therapeutic intervention that directly addresses this relationship. Therefore, an evidence-based approach to helping her cope with her anxiety better (thereby, potentially attenuating her physical symptoms) could be very helpful. RESPONSE: The most appropriate next step would be to (A) Recommend behavioral therapy. This patient's symptoms of frequency and incontinence are known as "nervous bladder" and are considered a functional urinary disorder often related to mental stress or anxiety. Cognitive-behavioral therapy, a type of behavioral intervention, has been shown to be effective in managing functional urinary disorders. This approach can help the patient to understand and mitigate her response to anxiety-provoking situations, potentially reducing the occurrence of her symptoms.

**Facilitator** The consensus among the AI responses is that the most appropriate next step in managing the 24-year-old woman's symptoms of increased urinary frequency and occasional incontinence, which appear to be triggered by stress or anxiety related to travel and examinations, is to recommend behavioral therapy. This recommendation is based on the understanding that her symptoms are likely a psychosomatic manifestation of anxiety, given the temporal relationship between the onset of symptoms and stressful events, and the absence of any physical abnormalities or medical conditions as indicated by her normal physical examination and laboratory results. Behavioral therapy, specifically cognitive-behavioral therapy (CBT), can equip her with strategies to manage her anxiety and potentially reduce her symptoms. Other interventions such as psychoanalytic psychotherapy, stress avoidance, reviewing her sexual history, or simply reassuring her that her symptoms will resolve over time, are considered less beneficial or practical in this case.
